# Photoactive Au@MoS_2_ Micromotors for Dynamic Surface-Enhanced Raman Spectroscopy Sensing

**DOI:** 10.1021/acsami.3c12895

**Published:** 2023-11-16

**Authors:** Víctor de la Asunción-Nadal, Juan Victor Perales-Rondon, Alvaro Colina, Beatriz Jurado-Sánchez, Alberto Escarpa

**Affiliations:** † Department of Analytical Chemistry, Physical Chemistry, and Chemical Engineering, Universidad de Alcala, Alcala de Henares, E-28802 Madrid, Spain; ‡ Department of Chemistry, University of Burgos, Pza. Misael Bañuelos s/n, E-09001 Burgos, Spain; § Chemical Research Institute “Andres M. del Rio”, Universidad de Alcala, E-28802 Madrid, Spain

**Keywords:** photophoretic, swarming, dichalcogenides, mobile SERS substrates, crystal violet, malachite green, paraquat

## Abstract

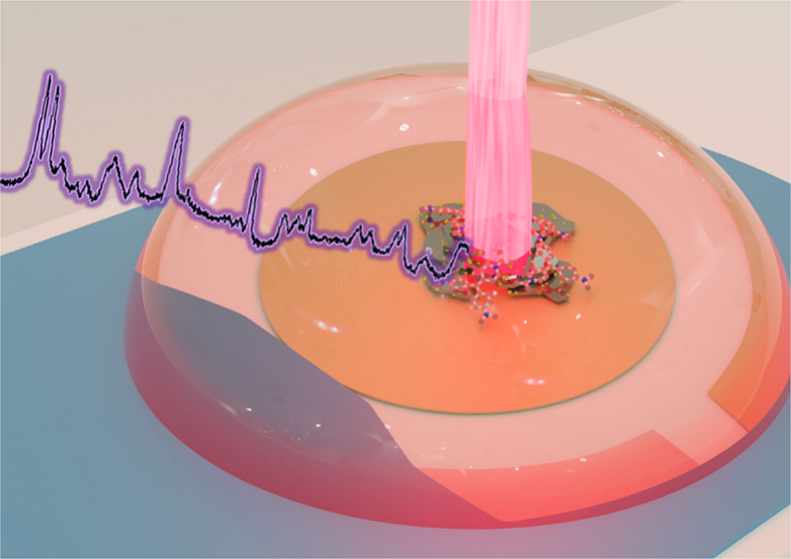

Photophoretic Au@MoS_2_ micromotors are used as smart mobile substrates for dynamic surface-enhanced Raman spectroscopy (SERS) sensing. The photophoretic capabilities and swarming-like propulsion of the micromotors allow for their schooling and accumulation in the measuring spot, increasing the density of SERS-active gold nanoparticles for Raman mapping and, simultaneously, the preconcentration of the target analyte. The generation of “hot-microflake spots” directly in the Raman irradiation point results in a 15–18-fold enhancement in the detection of crystal violet without the requirement for additional external sources for propulsion. Moreover, the reproducible collective micromotor motion does not depend on the exact position of the laser spot concerning individual micromotors, which greatly simplifies the experimental setup, avoiding the requirements of sophisticated equipment. The strategy was further applied for the detection of malachite green and paraquat with a good signal enhancement. The new on-the-move-based SERS strategy holds great promise for on-site detection with portable instrumentation in a myriad of environmental monitoring and clinical applications.

## 1 Introduction

Surface-enhanced Raman spectroscopy (SERS) is a valuable technique for the direct detection of analytes or materials characterization, with wide applications forbiosensing and (bio)-imaging.^1^ The selection of appropriate plasmonic SERS substrates can amplify Raman signals up to 1 × 10^15^ times.^2−4^ Among the materials with SERS activity, gold,^5,6^ silver,^7^ and copper^8^ with different nanostructures and morphologies are the most promising. Although other materials have shown promising SERS-based activity,^9^ recent advances are also aimed at the combination of high-surface materials such as carbon-based paper and graphene, among others, with SERS-active materials, allowing for a previous enrichment of the analyte following SERS detection.^10−12^

Different approaches have been proposed to combine the potential of SERS-based detection with other existing promising technologies. Among them, the use of microfluidic platforms^13^ or the combination of wire-in-cavity-in-bowl structures^14^ for SERS sensing has been recently explored in the literature. Yet, the incorporation of micromotors as platforms for reliable SERS detection has been scarcely exploited.

Micromotors are microstructures capable of converting an energy input, such as chemical, mechanical, magnetic, or electromagnetic, into movement.^15−18^ The ability of micro- and nanomotors to perform on-demand tasks on the microscale has been exploited in active SERS sensing approaches. Rolled-up Au/SiO/Ti/Ag micromotors propelled by peroxide fuel have been used for dynamic capture of Rhodamine 6G as a model analyte, followed by SERS detection.^19^ Yet, the requirements for peroxide fuel restrict the detection of in vitro schemes in nonbiological samples. To increase the biocompatibility for application in raw biological samples, magnetic rolled-up Au/SiO/Fe micromotors can be used with a moderate SERS increase.^20^ Yet, an additional external source (magnetic generation setup) for propulsion is needed. Phototactic Ag@SiO_2_ micromotors can experience self-diffusiophoresis by the action of UV light irradiation, quickly aggregating and enhancing the SERS signal. The strategy was applied for crystal violet (CV) and MCF-7 cancer cell detection, enhancing the signal 6 and 3 times, respectively.^21^ While advantageous, the main drawbacks of such a procedure are the requirement for external sources (UV lamps) for micromotor propulsion along with the limited application in salt-rich environments, preventing micromotor motion. Helical magnetic micromotors have been applied for intracellular SERS biosensing in HeLa cells.^22^

Herein, we describe a SERS strategy using photophoretic Au@MoS_2_ micromotors. Unlike previous micromotor-based SERS strategies, our work presents unique advantages such as micromotor motion with the same laser source used for Raman detection in the absence of peroxide or surfactants, avoiding external motion sources. Additionally, our micromotors can move in salt-rich environments due to the unique photophoretic collective motion, allowing its application in complex biological samples and other complex matrices. In the following sections, we will illustrate the capabilities of this versatile, dynamic SERS-based sensing micromotor approach toward the analysis of light and nonlight-responsive molecules.

## 2 Results and Discussion

**Figure 1:**
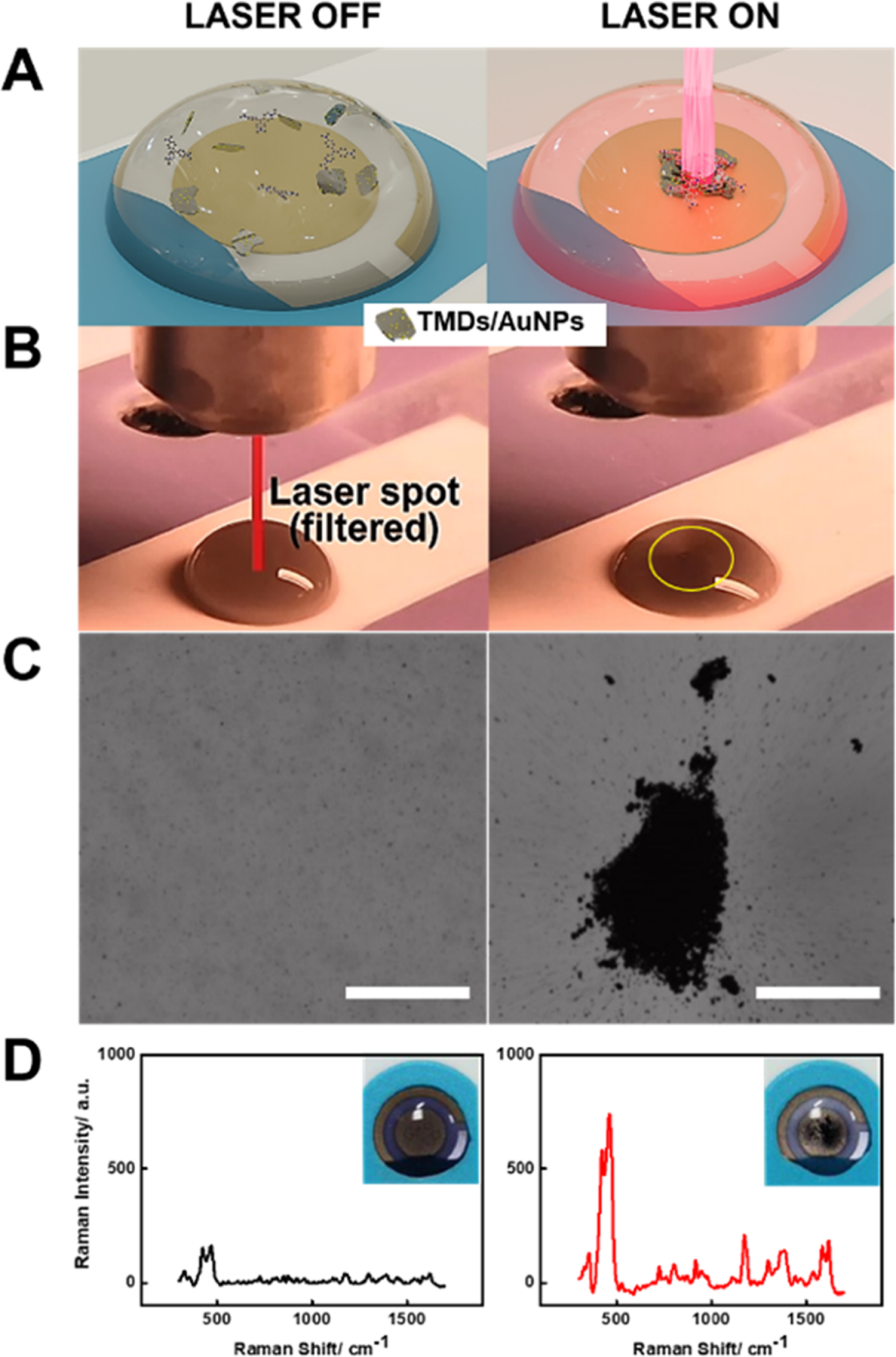
(A) Schematic of the Au@MoS_2_ micromotors for dynamic SERS sensing prior to and after irradiation with the laser. (B) Time-lapse images (taken from Video S1) of the drop before and after turning the laser ON. (C) Time-lapse images taken using an external camera and an optical microscope. Scale bar: 50 μm. (D) SERS spectra of crystal violet with Au@MoS_2_ micromotors at *t* = 0 s (black) and *t* = 1000 s (red). Insets show the images of the surface under each condition.

The concept of the Au@MoS_2_ micromotors for SERS enhancement/dynamic sensing is depicted in Figure 1. The Au@MoS_2_ micromotors are prepared by exfoliation of the material, followed by *in situ* decoration with gold nanoparticles by the spontaneous reduction of tetrachloroauric acid (HAuCl_4_) onto the MoS_2_ surface, generating efficient SERS substrates. The resulting composites display photophoretic capabilities, and the propulsion and swarming can be thus triggered by irradiating the sample with the incident laser. This will, in turn, translate into a dual effect, as it will allow the micromotor accumulation to harvest the target analytes and ultimately concentrate in the measuring spot, increasing both the density of SERS-active gold nanoparticles and concomitantly analyte preconcentration.

Our approach presents some major advantages compared to previously reported rolled-up or Ag@SiO_2_ micromotors,^19−21^ such as the use of the same laser for both inducing the movement of the micromotors and their accumulation and allowing the analyte to be dynamically measured by registering the Raman signal. This greatly simplifies the setup, as further light sources must not be aligned and focused. Moreover, due to the long-range effect of the photophoretic mechanism compared to the photocatalytic approach, there is no need to aim at a single micromotor, thus allowing for the use of simpler and cheaper instrumentation such as low-resolution Raman setups. In this way, a more reproducible motion is achieved, as the number of involved micromotors is higher (swarming phenomenon) and does not depend on the exact position of the spot relative to that of the micromotors. Combining these features, the following approach can provide 18-fold increased signals in CV detection, as reflected in the SERS spectra of Figure 1D.

**Figure 2:**
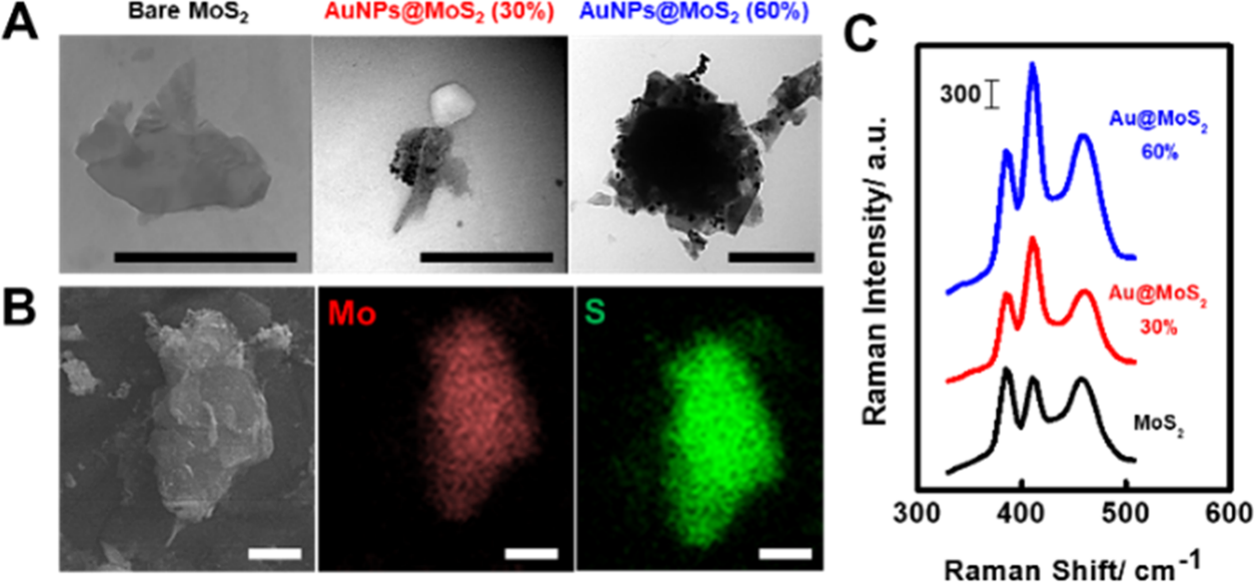
(A, B) TEM and SEM/EDX characterization of Au@MoS_2_ micromotors for dynamic SERS sensing. (C) SERS of bare MoS_2_ (black), 30% Au@ MoS_2_ (red), and 60% Au@ MoS_2_ (blue). Scale bars: 500 nm (TEM) and 1 μm (SEM).

Particularly, in semiconductors such as MoS_2_ under suitable light irradiation, electron–hole pairs are generated. If the relaxation occurs in a nonradiative manner and is instead mediated by phonons, an increase in the lattice temperature is noted.^23,24^ Indeed, in the case of suspended micrometer-sized particles, these localized temperature gradients may generate a directional motion toward (positive photophoresis) or against (negative photophoresis) the light spot. Such photothermal-induced motion has been previously reported, allowing for the focusing of photophoretic particles in a UV light spot.^25,26^ Additionally, our research group has described and modeled such photophoretic collective behavior for WS_2_ microflakes.^27^ Hence, in this report, the introduction of gold nanoparticles as active plasmonic materials to enable the use of photophoretic MoS_2_ micromotors as smart accumulating SERS substrates and subsequent analyte harvesting will be explored. To obtain the best SERS performance, first, the micromotor synthesis was optimized to incorporate the highest amount of gold nanoparticles without hampering the efficient propulsion. As can be seen in Figure 2A, transmission electron microscopy (TEM) images show that the modification was successful, with the gold nanoparticles homogeneously distributed along the surface of the micromotors. Nanoparticles were measured with mean sizes of 9 ± 2 nm for 30% mol/mol gold content and 27 ± 5 nm for 60% mol/mol Au/Mo content (for high-magnification TEM images, please see Figure S1). The chemical composition of both microcomposites was further assessed by scanning electron microscopy (SEM) and energy-dispersive X-ray (EDX) mapping, which shows adequate element distribution (see Figure 2B and Table S1). Well-defined signals corresponding to the three main Raman bands for MoS_2_ were obtained.^28,29^

Further, Raman characterization illustrates the modification, with marked changes in the MoS_2_ A_1g_ band (410 cm^–1^) as the amount of AuNPs increases (see Figure 2C). It is also important to note differences in the relative intensities of the main bands of the MoS_2_ substrate. In the case of both 30 and 60% Au@MoS_2_, the relative intensity of the A_1g_ band is increased compared to the other main bands, having different proportions from that of the unmodified material. In addition, there is a net increase of the Raman intensity in all bands, which is expected due to the SERS effect promoted by the AuNPs deposited on it. Band assignment is given in Table S2 in the Supporting Information.

**Figure 3:**
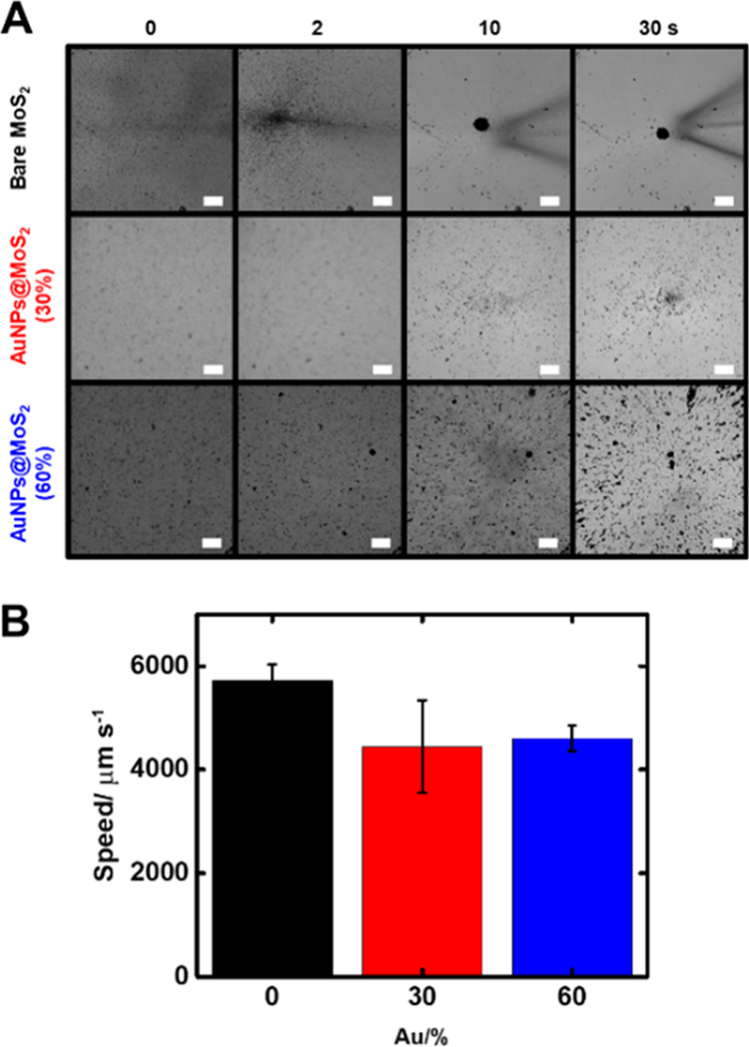
(A) Time-lapse images (taken from Video S2) over time of bare MoS_2_, 30% Au@ MoS_2_, and 60% Au@ MoS_2_ micromotors. Scale bars: 50 μm. (B) Corresponding speed plots.

After characterizing the Au@MoS_2_ micromotors and assuring the efficient modification with the substrate, we evaluated the effect of gold nanoparticle content on the motion and swarming behavior of the micromotors and their future role in SERS sensing. Figure 3 illustrates the time-lapse images (taken from Video S2) of bare MoS_2_ and 30 or 60% gold nanoparticle content in Au@MoS_2_ micromotors at different times. To carry out this experiment, we reproduced the Raman setup conditions using an inverted optical microscope and a light-emitting diode (LED) source to irradiate the micromotors in the near-infrared region (700 nm). As can be seen in Figure 3A, prior to irradiation with light, the micromotors are dispersed in the solution, exhibiting Brownian motion (OFF state). After activation of the laser (irradiation source), the micromotors start experiencing a swarming behavior (ON state), navigating the solution (and preconcentrating the analytes) toward the laser spot, resulting in the accumulation after 30 s irradiation. Such fast swarming and schooling behavior that results in the generation of an accumulation spot is due to a photophoretic effect. If the laser is turned off, the micromotor cluster gets disassembled, and the micromotors are dispersed in solution by Brownian and electrostatic interactions. In brief, upon light irradiation, the micromotors heat up by the action of the material and excitation and promotion of electrons in the electronic structure of the material. Subsequently, heat dissipation in the solvent induces a hydrodynamic flow that draws the micromotors into the spot as a photophoretic swarm.^30^ To check the potential effect of electrophoretic or self-diffusiophoretic mechanisms, in a previous report, we checked the propulsion of the micromotors in *N*,*N*-dimethylformamide, since in this media, radical production essential for electrophoretic flow generation is prevented.^30^ Similar speeds were observed in both media; thus, the main mechanism responsible for propulsion is the photophoretic effect. As such, the micromotors display two motion behaviors: a fast convective motion and crawling toward the focal point/laser beam in the ON state and Brownian motion and electrostatic-driven repulsion in the OFF state, with results in the diffusion of the micromotors away from the accumulation point. Additionally, if a different spot is irradiated, the micromotors can move as a swarm to the new focal point, clearing the original spot. As such, the motion of the micromotors can be controlled by turning the laser ON–OFF or moving the laser point during the measurements.^27^ It is also important to study the effect of the irradiation time on the SERS signal, as will be further illustrated. Additionally, the speed plots of Figure 3B illustrate the fast propulsion of the micromotors, reaching speeds of up to 6 mm s^–1^. After modification with gold nanoparticles, a slight decrease in the speed to 5 mm s^–1^ is noted, which, however, does not hamper the motion or efficient swarming behavior of the micromotors, showing yet a remarkably high speed. Also, the decrease in speed is not significant, and it does not prevent the success of the analytical operation: the accumulation of the micromotors and the subsequent detection of the analyte on the SERS substrates on board the micromotors. This nonsignificant effect of the decrease in speed can be due to the decrease in the available content of the photophoretic MoS_2_ material in the micromotor by mass, hence attenuating the light-induced motion capabilities. Notably, the speed of the photophoretic micromotors differs significantly between those that navigate in the bulk of the solution (higher speed) and those that navigate near the microscope substrate (lower speed) where they are irradiated. As previously reported by our group,^31^ when the micromotors are in close contact with the microscope substrate (glass slide), micromotor–substrate interactions arise, hindering the motion. In Figure 3A, the interactions of the micromotors with increasing amounts of AuNPs become apparent. As expected, the heavier micromotors with higher amounts of AuNPs show bigger micromotor–substrate interactions. However, this effect is less relevant in the bulk, where the speed was recorded with characterization and comparative purposes in Figure 3B. This difference in the behavior of the micromotors can be seen in Video S2.

**Figure 4:**
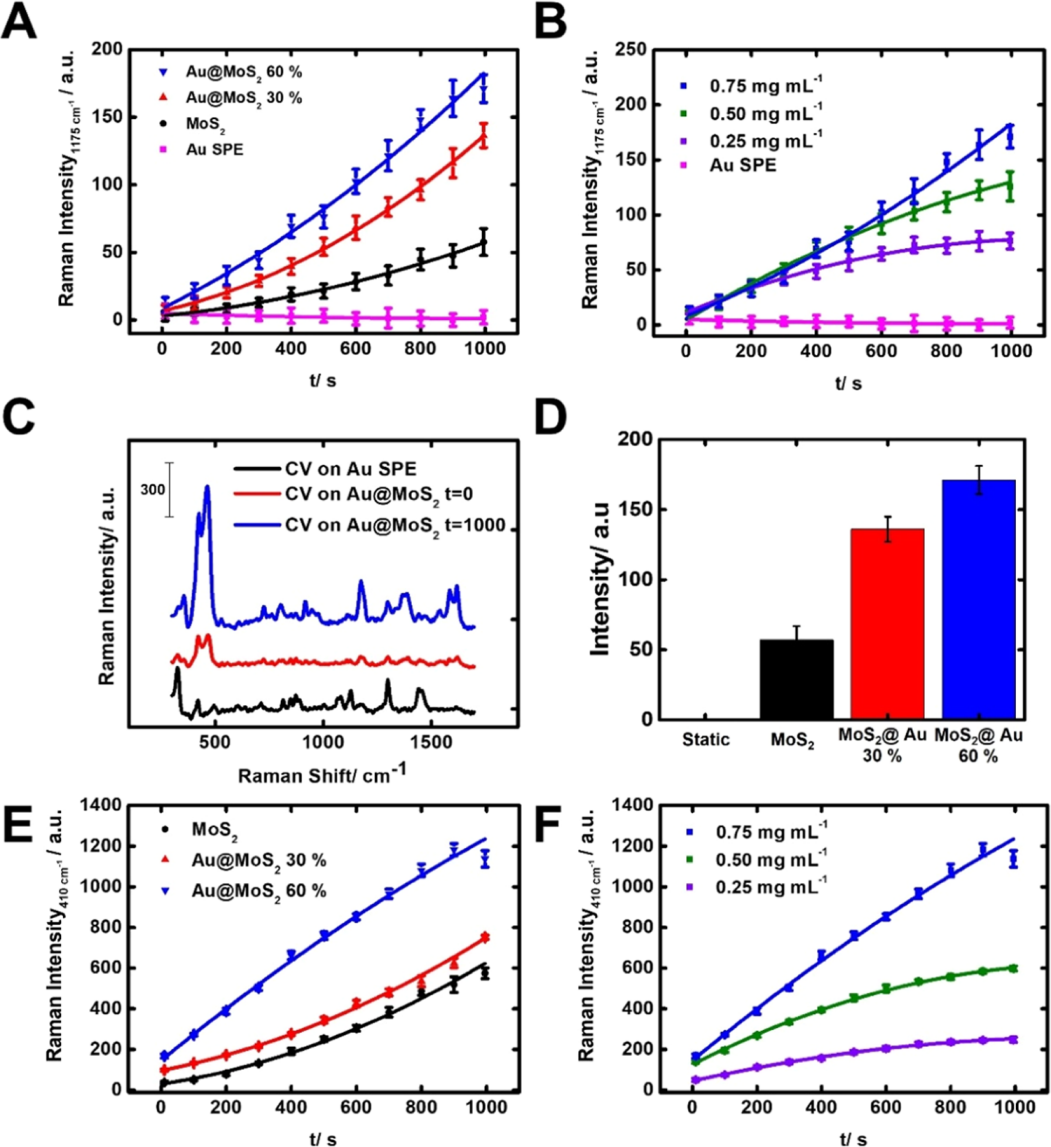
Characterization of the SERS enhancement process with the micromotors on a gold screen-printed surface. (A) CV SERS signal over time without the SERS substrate (gold screen-printed surface) (pink), MoS_2_ without gold nanoparticles (black), and MoS_2_ with 30% (red) and 60% (blue) molar gold content. (B) CV SERS signal over time with different amounts of 60% Au@MoS_2_. (C) SERS spectra of CV over time under the optimized conditions. (D) Corresponding intensity of the 1175 cm^–1^ peak for static, MoS_2_ without gold nanoparticles (black), and MoS_2_ with 30% (red) and 60% (blue) molar gold content. (E) MoS_2_ A_1g_ (410 cm^–1^) SERS signal over time of unmodified MoS_2_ micromotors (black) and MoS_2_ with 30% (red) and 60% (blue) molar gold content. (F) MoS_2_ A_1g_ SERS signal over time with different amounts of 60% Au@MoS_2_ micromotors.

Once we tested the successful modification of the MoS_2_ micromotors with gold nanoparticles and the efficient propulsion, we studied the performance of the micromotors for SERS enhancement in the detection of a wide range of molecules, including CV, malachite green (MG), and paraquat. CV was used as a probe molecule to characterize the SERS enhancement phenomena, given its recognizable SERS spectrum and its high Raman cross section (see band assignment in Table S3).^32−34^ The results are listed in Figure 4.

We studied first the effect of the weight content of Au nanoparticles on the micromotors. As shown in Figure 4A, the micromotors with 30 and 60% gold content show a 2.5- and 3.5-fold increased signal, respectively, compared to bare MoS_2_ when they are used as a SERS substrate for 1000 s. This justifies the use of plasmonic gold nanoparticles in the microcomposite. Additionally, a 15–18-fold increase is observed with micromotors with 30 and 60% gold nanoparticle content compared to static micromotors (*t* = 0, no motion), thus illustrating the effect of light-induced harvesting and preconcentration. Specifically, the reported micromotors play a dual role in SERS enhancement. First, the gold-modified micromotor acts as a SERS substrate, enhancing the signal of the target molecules. Second, due to the light-induced accumulation, this SERS substrate is preconcentrated from the bulk to the irradiated area. As more SERS substrate is brought to the area where the SERS signal is recorded, the signal increases. It is also important to note that the micromotors perform harvesting of the analyte, as they travel to the focal point, as noted by the decrease in the intensity of the solution (data not shown). This finding is relevant, as not only is the substrate accumulated, but the analyte is also harvested and preconcentrated in the measurement region. The combination of the reported effects contributes to the observed signal enhancement. It is worth noting that a greater preconcentration effect is found for these Au@MoS_2_ micromotors compared with those composed of only MoS_2_.

As can be observed from Figure S2, the ratio of Raman intensity at 1175 cm^–1^ (model analyte CV, band)/Raman intensity at 410 cm^–1^ (micromotor band) as a function of time shows that MoS_2_ micromotors have a constant value in time, which means that the increase of signal for both the CV molecule/sample (1175 cm^–1^) and the micromotors/MoS_2_ (410 cm^–1^) has the same trend, and no additional enhancement, out of that one due to the accumulation of micromotors, is observed. However, when we observe the values for the Au@MoS_2_ micromotors, the ratio increases in time for both tested Au@MoS_2_ micromotors due to an increase of the CV molecule (1175 cm^–1^) band. The latter means that an additional enhancement is found for Au@MoS_2_ micromotors, which is additive to the existing accumulation of the micromotor effect. We attribute this effect to the preconcentration of the molecule on the gold nanoparticles deposited on the MoS_2_ surface, since they are active on these substrates.

Notably, the light-induced accumulation effect is more noticeable in the case of 30% gold content micromotors, as their initial SERS effect is lower and their motility is higher. Remarkably, in either case, the light-induced motion increases notably the SERS effect. Next, we studied the effect of the amount of micromotors, selecting 60% Au@MoS_2_ due to the higher output signal. Different amounts of micromotors ranging from 0.75 to 0.25 mg mL^–1^ (mass/volume) were tested. As expected, increasing the amount of substrate translates into a gradual increase in the signal (see Figure 4B). Thus, a content of 0.75 mg mL^–1^ micromotors was selected as optimal. Under the optimized conditions, we recorded the entire spectrum of CV, as depicted in Figure 4C. The role of micromotor movement in the enhanced detection was reflected in the fact that without the addition of micromotors, no signal was observed. Additionally, the CV SERS signal is very weak at *t* = 0 in the presence of the micromotors. Nonetheless, after 1000 s irradiation, the typical spectra of CV were observed, with peaks at 1175 cm^–1^ (C–H bond bending), along with 1367 cm^–1^ (from the *N*-phenyl bond) and 1618 cm^–1^ (C–C bond stretching of the benzene ring).^32^ The signal intensity at 1175 cm^–1^ was plotted at each condition in Figure 4D. As can be seen, a 15-fold increase is obtained using the 60% Au@MoS_2_ micromotors compared with the nonirradiated micromotors, illustrating the suitability of the new micromotor-based approach of the enhancement of the SERS signal.

To get further insights into the schooling and aggregation processes of the micromotors, the signal of the A_1g_ band of MoS_2_ (410 nm) was recorded for unmodified micromotors and micromotors with 30 and 60% gold content over time, as well as different concentrations of 60% Au@MoS_2_ micromotors. As can be seen in Figure 4E, in all cases, an increase in the signal of MoS_2_ is noted. This denotes a consistent accumulation of both the AuNPs-modified and unmodified micromotors throughout the experiment’s duration. As expected, the magnitude of the Raman intensity is higher in the case of micromotors modified with gold nanoparticles. To properly interpret the results, the SERS effects of AuNPs must be taken into consideration. Hence, the higher Raman output might be due to a higher SERS activity, as the same amount of micromotors is used in every case. This is in line with the results found in Figure 4A. As can be seen in Figure 4F, by comparing the A_1g_ band of MoS_2_ of different 60% Au@MoS_2_ micromotor concentrations, we can observe an increasing Raman signal at *t* = 1000 s with higher amounts of micromotors. This indicates the accumulation of higher amounts of micromotors in the laser spot. The observed trend matches that observed in Figure 4E, revealing the importance of light-enabled motion and accumulation of Au@MoS_2_ substrates. Interestingly, the signal at *t* = 0 s for MoS_2_ is relatively higher than the signal of CV at the starting time. This observation emphasizes the importance of the analyte harvesting observed in the sample, as depicted in Figure 1D. Overall, the results reported in this manuscript reveal an enhancement of the SERS signal of a model analyte that depends directly on the light-driven actuation of the micromotors and their subsequent accumulation on the laser spot.

**Figure 5:**
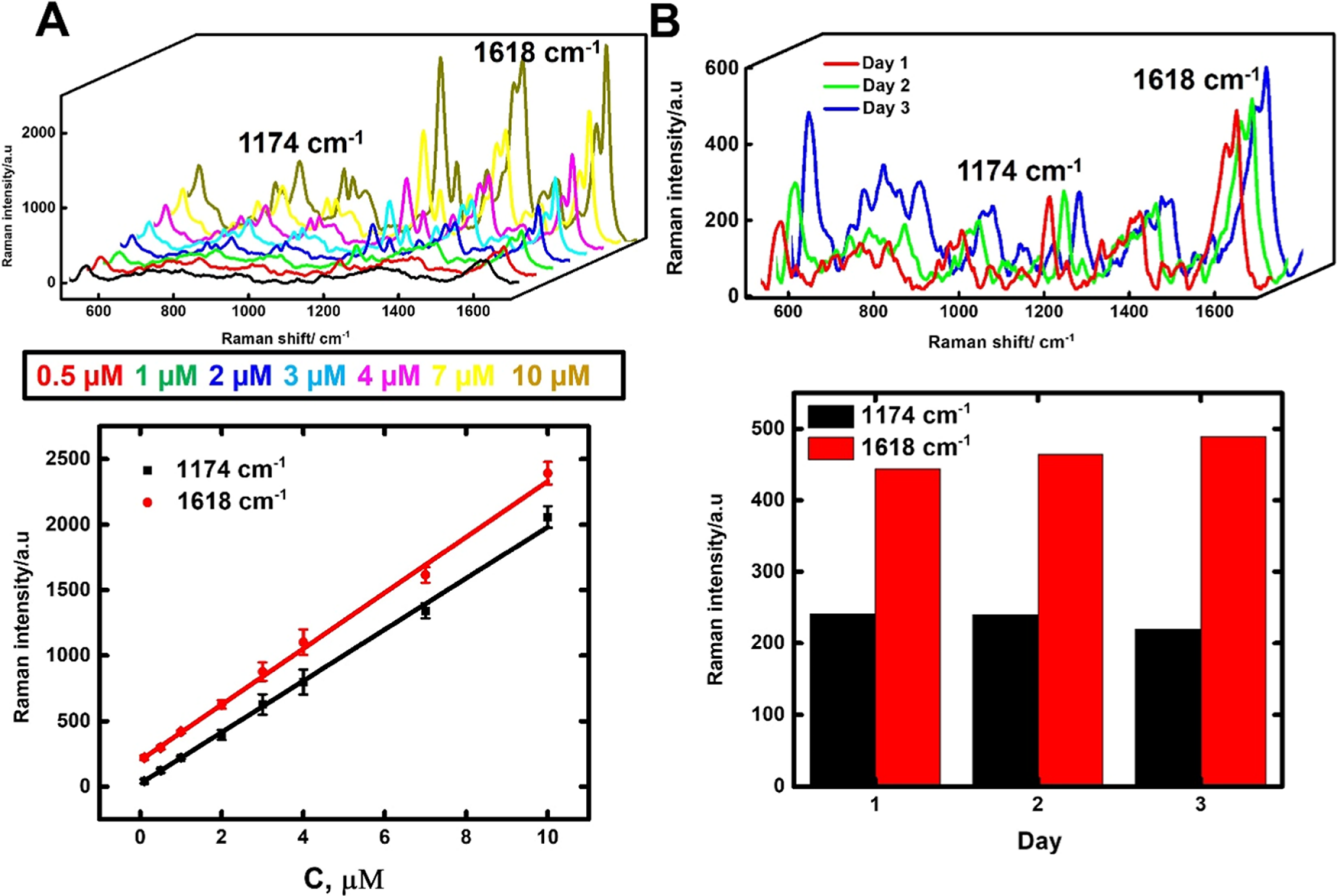
On-the-move SERS micromotor-based approach for the detection of MG. (A) SERS spectra of different concentrations of MG (top) and corresponding calibration plots using the main signals at 1174 and 1618 cm^–1^ at 350 s. (B) Precision of the approach: SERS spectra obtained from the measurements of MG (1 μM) on three different days (top) and corresponding Raman intensities at 1174 and 1618 cm^–1^ (bottom). Error bars show the standard deviation of three measurements.

To test the practical applicability of the micromotors, several analytes were tested, and the analytical performance was evaluated through calibration plots, precision studies, and recovery percentages. Figure 5 shows the results obtained in the measurement of MG, which is an important aquatic compound for controlling and healing fish diseases. However, due to its hazardous effect, the use of this pesticide has been banned in aquiculture. Therefore, developing accurate and reliable analytical approaches is vitally important for monitoring residual hazardous chemicals in water and fish tissues. Please note that even after being an absorbing molecule in the visible region, the absorption spectrum of MG as well as CV is far from the wavelength of the laser (785 nm), which prevents any resonance in the Raman response.^35,36^ Therefore, no influence of the laser on the studied molecules is expected. As can be seen in Figure 5A, the characteristic Raman bands of the target compound are observed (see also Table S4). The two main Raman shifts at 1174 and 1618 cm^–1^ were selected to construct the calibration plot, extracting the Raman signal at 350 s of the beginning of the experiment. Linear responses were obtained over the range of 0.3–10 μM in all cases (*r* = 0.999), with a detection of 0.1 μM. No response was obtained in the absence of moving micromotors (not shown), revealing again the crucial role of movement in preconcentration/analyte detection. To check the reproducibility of the Raman signals during the measurements, we studied the intermediate precision of the approach (*n* = 3 days). As can be seen in Figure 5B, the signals are highly reproducible, with a relative standard deviation of 5%. This fact reveals the uniform accumulation of the Au@MoS_2_ materials over time and between different experiments due to the same amount of micromotors used during the experiments (0.75 mg mL^–1^, see Figure 4B,F for the optimization). To further test the practical applicability, we carried out an analysis of tap water. Test samples were collected directly from the regular water supply without any further treatment, and subsequently, they were fortified with 1.5 μM of MG, obtaining a recovery percentage of 112 ± 5%.

**Figure 6:**
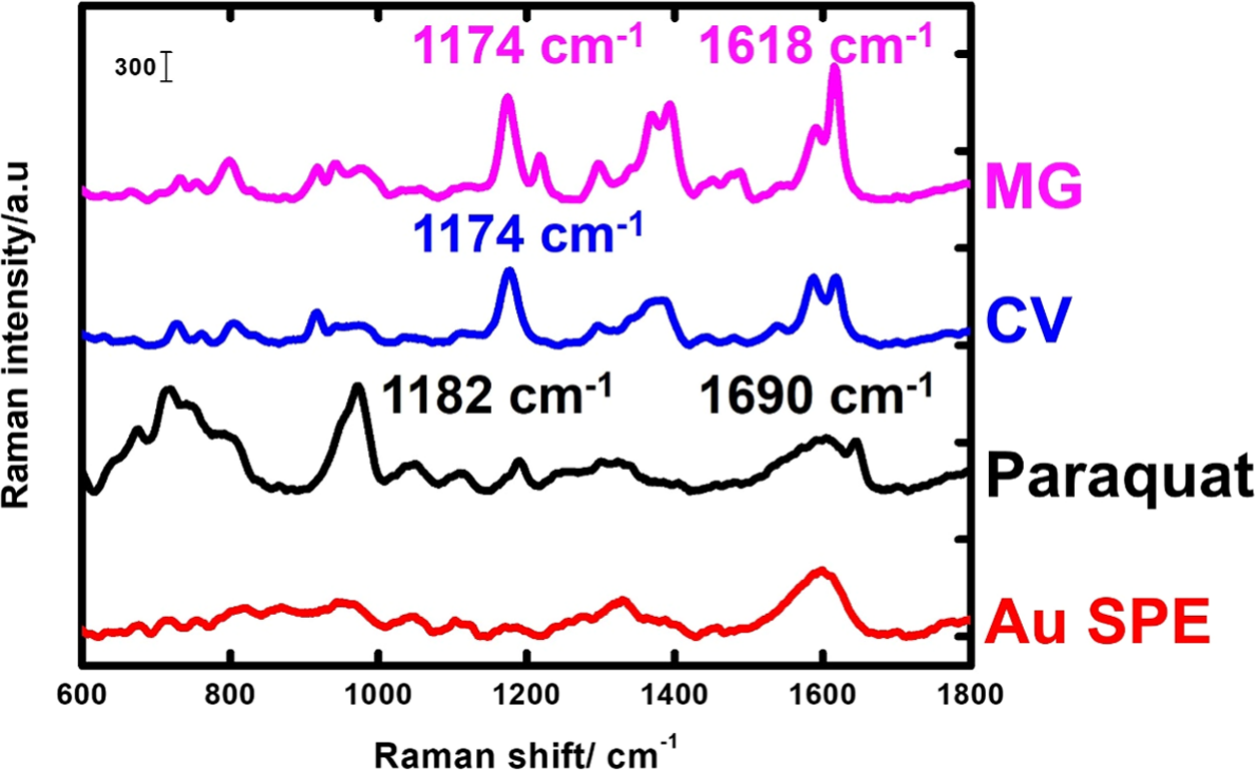
SERS spectra of MG (15 μM), CV (5 μM), and paraquat (5 μM) using the micromotor-based dynamic sensing approach and control experiment without micromotors on the gold screen-printed surface.

Additionally, the SERS micromotor approach was tested for the detection of another compound such as paraquat, which does not present absorption in the visible region of the spectrum with very similar results. Excellent signals with identifiable peaks were obtained, as depicted in Figure 6, showing the SERS spectra for all of the studied molecules.

## 3 Conclusions

Au@MoS_2_ micromotors have been demonstrated to be highly efficient substrates for dynamic SERS sensing. The micromotors experience controlled photophoretic-based swarming behavior upon irradiation with the laser source. This results in a fast accumulation of the gold nanoparticles-decorated micromotors in a spot to greatly increase the SERS signal, resulting in a 15–18-fold enhancement in the detection of CV compared with the nonirradiated micromotors. Analytical capabilities of the micromotor-based dynamic sensing approach were demonstrated toward the detection of relevant compounds in the water quality assessment of different natures, MG and paraquat; both are light and nonlight responsive. One of the main drawbacks of SERS detection in micromotor-based approaches is related to the need to focus the laser spot on the swimming motors. In this work, we demonstrate that the micromotors swim to the laser beam focus, simplifying the detection while the target molecule is additionally collected and preconcentrated. The micromotors do not require fuel or additional reagents for propulsion and smartly swim to the laser focus, thus representing a universal platform that holds considerable promise for SERS dynamic sensing in just a drop, especially for cheap and simpler Raman setups. The versatile label-free approach could be adapted to other biofunctionalization routes for the highly selective detection of a myriad of analytes. Future efforts should be aimed at testing the strategy in complex media such as blood in the detection of complex (bio)analytes or at combining the micromotors with other substrates such as silver or copper for enhancing SERS sensing.

## 4 Experimental Section

### 4.1 Reagents and Materials

All chemical agents were purchased from Merck Co., Ltd., at the highest purity available. Gold(III) chloride trihydrate (cat. G4022), molybdenum disulfide (cat. 234842), potassium nitrate (cat. 221295), CV (cat. C6158), paraquat (cat. 36541) and MG (cat. 38978) were used as received without further purification. All solutions were prepared using ultrapure water obtained from a Millipore DirectQ purification system provided by Millipore (18.2 MΩ cm resistivity at 25 °C).

### 4.2 Synthesis of Au@MoS_2_ Micromotors

A vial containing 0.75 mg mL^–1^ MoS_2_ in ultrapure water was sonicated using a tip sonicator for 1 h or until the micromotors displayed phototaxis. Next, a concentrated HAuCl_4_ solution was added dropwise while being sonicated according to the final gold content (30 or 60% molar). The resulting material was centrifuged, resuspended in water, and stored until further use. Before every experiment, the modified Au@MoS_2_ and unmodified micromotors were sonicated for 0.5 h or until the phototaxis properties were restituted.

### 4.3 Characterization of Au@MoS_2_ Micromotors

A Jeol JSM 6335F scanning electron microscope was used to characterize the prepared Au@MoS_2_ materials. Images were recorded with the secondary electron detector and using an accelerated voltage of 15 kV. The EDX mapping analysis to obtain a map of the elemental composition of the microtubes was carried out using an Oxford Instruments, model: X-Max de 80 mm^2^ with a resolution of 127 eV–5.9 keV. Transmission electron microscopy (TEM) images were taken using a Zeiss M-10 microscope. TEM pictures of micromotors containing different amounts of gold nanoparticles were analyzed by using ImageJ software.

### 4.4 Raman Measurements

SERS experiments were carried out on gold screen-printed electrodes (220 BT, Metrohm Dropsens). A 5 μM crystal violet solution was used as a test sample. Malachite green and paraquat stock solutions were prepared in water at desired concentrations. Typically, 5 μL of micromotors were mixed with a specific volume of the sample, and then, 50 μL of this solution was transferred to the electrode. Time-resolved Raman spectroscopy experiments were performed by using a customized SPELEC RAMAN instrument (Metrohm DropSens), which integrates a laser source of 785 nm. Laser power in all experiments was 80 mW (254 W·cm^–2^). This instrument was connected to a bifurcated reflection probe (DRP-RAMANPROBE, Metrohm DropSens). A Raman spectroelectrochemical cell, especially designed for screen-printed electrodes, was employed. DropView SPELEC software (Metrohm DropSens) was used to control the instrument and process the data collected in each experiment. Raman frequencies were calibrated using a Si wafer (520.6 cm^–1^) before the experiment. Likewise, before a set of experiments, the focusing of the probe was carried out by optimizing the Raman signal of the Si wafer.

## Supporting Information Available

Element analysis by EDX of the different MoS_2_ micromotors; band assignment for the SERS spectrum of crystal violet; transmission electron microscopy images of gold nanoparticles on MoS_2_ flakes; and ratio of Raman intensity at 1175 cm^–1^/Raman intensity at 410 cm^–1^ versus experimental time (PDF)
Irradiation of the micromotor drop with the laser of the Raman equipment (to acquire the video, an optical filter was used to remove the laser signal to better observe the accumulation of the material) (Video S1) (MP4)
Propulsion of MoS_2_, 30% Au@MoS_2_, and 60% Au@MoS_2_ micromotors under light irradiation (Video S2) (MP4)
