# Supplementary material for: Photoactive Au@MoS2 Micromotors for Dynamic Surface-Enhanced Raman Spectroscopy Sensing
Source: ACS Appl Mater Interfaces. 2023 Nov 16;15(47):54829–37. doi: 10.1021/acsami.3c12895 (PMC10694815; doi:10.1021/acsami.3c12895)
Supplement: Supplementary file 1 — am3c12895_si_001.pdf [file am3c12895_si_001.pdf]

## Supporting Information

**Photoactive Au@MoS$_2$ micromotors for dynamic surface-enhanced Raman spectroscopy sensing**

Víctor de la Asunción-Nadal,[a] Juan Victor Perales-Rondon,[a,b] Alvaro Colina,[b] Beatriz Jurado-Sánchez,*[a,c] and Alberto Escarpa*[a,c]

[a]Department of Analytical Chemistry, Physical Chemistry, and Chemical Engineering, Universidad de Alcala, Alcala de Henares, E-28871 Madrid. beatriz-jurado@uah.es; alberto.escarpa@uah.es

[a]Department of Chemistry, University of Burgos, Pza. Misael Bañuelos s/n, E-09001 Burgos, Spain.

[c]Chemical Research Institute "Andres M. del Rio", Universidad de Alcala, E-28807, Madrid, Spain.

**Table S1.** Element analysis by EDX of the different MoS$_2$ micromotors.

| | MoS$_2$ | 30 % Au@MoS$_2$ | 60 % Au@MoS$_2$ |
|---|---|---|---|
| Mo | 61 | 30.6 | 34.3 |
| S | 39 | 32.6 | 23.3 |
| Au | 0 | 36.8 | 42.4 |

**Table S2.** Band assignment for the SERS spectrum of MoS$_2$.1,2

| **Raman shift / cm -1** | **Vibrational assignment** |
|---|---|
| 384 | $\mathrm{E}^1_{2g}$ |
| 410 | $A_{1g}$ |
| 457 | $2LA$ |

**Table S3.** Band assignment for the SERS spectrum of crystal violet.[3–5]

| Raman shift / $cm^{-1}$ | Vibrational assignment |
|---|---|
| 1175 | C–H in plane bending |
| 1294 | C-C stretching |
| 1367 | N-phenyl stretching |
| 1388 | N-phenyl stretching |
| 1540 | C–C stretching |
| 1585 | C–C stretching |
| 1618 | C–C stretching |

**Table S4.** Band assignment for the SERS spectrum of malachite green.[6]

| Raman shift / $cm^{-1}$ | Vibrational assignment |
|---|---|
| 798 | C–H out-of-plane bending |
| 916 | C–H out-of-plane bending |
| 1174 | C–H in plane bending |
| 1618 | C-C stretching |

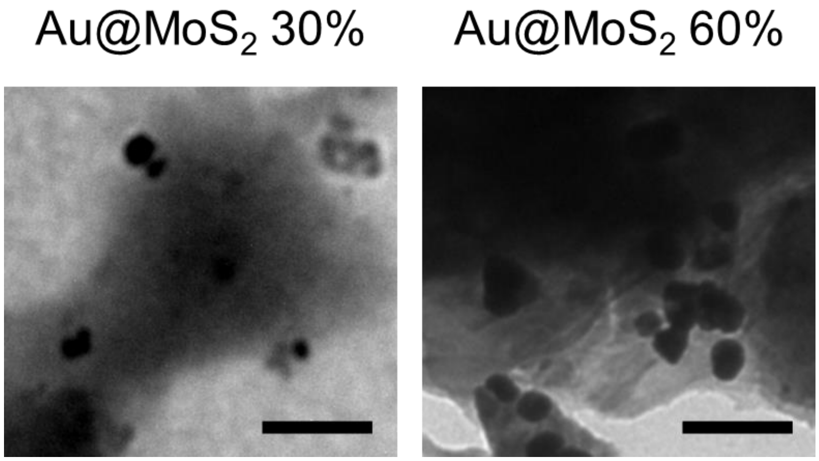


**Figure S1.** Transmission electron microscopy images of gold nanoparticles on $MoS_2$ flakes. Note that the nanoparticles show a spherical morphology. The mean size of the nanoparticles is 10 ± 2 nm (Au@$MoS_2$ 30%) and 23 ± 2 nm (Au@$MoS_2$ 60%). Scale bars 100 nm.

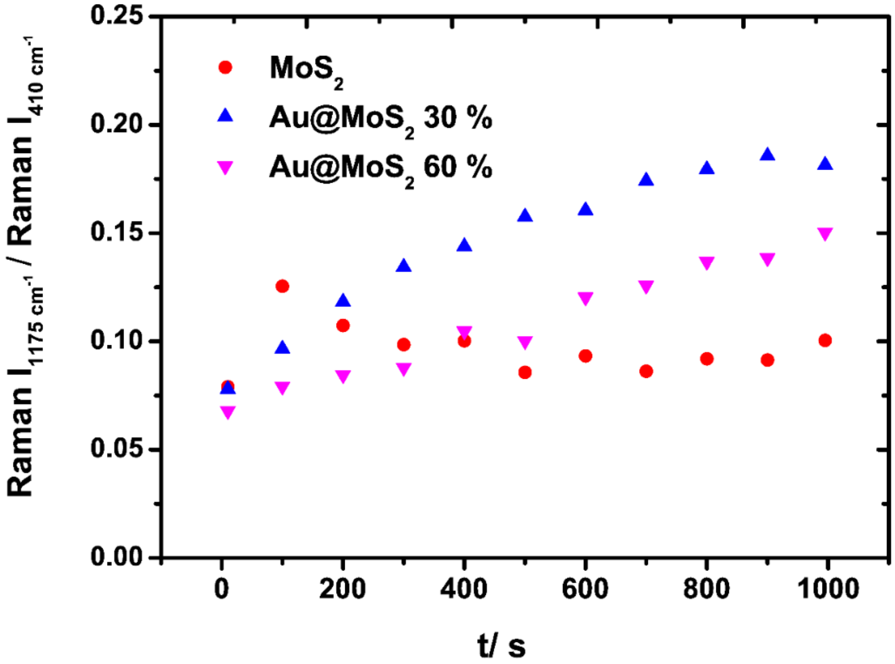


**Figure S2.** Ratio of Raman Intensity at 1175 $cm^{-1}$/ Raman Intensity at 410 $cm^{-1}$ vs experimental time. The data are taken from experiments in Figure 4 in the manuscript. In the plot are represented the micromotors composed of only $MoS_2$ (red circles), Au@$MoS_2$ 30 % (blue triangle) and Au@MoS2 60 % (purple triangle).

## References


1. Parkin, W. M.; Balan, A.; Liang, L.; Das, P. M.; Lamparski, M.; Naylor, C. H.; Rodríguez-Manzo, J. A.; Johnson, T. T. C.; Meunier, V.; Drndić, M. *ACS Nano* **2016**, *10*, 4134–4142.
2. Li, H., Zhang, Q.; Yap, C. C. R.; Tay, B. K.; Edwin, T. H. T.; Olivier, A.; Baillargeat, D. *Adv. Funct. Mater.* **2012**, *22*, 1385–1390.
3. Kleinman, S. L.; Ringe, E.; Valley, N.; Wustholz, K. L.; Phillips, E.; Scheidt, K. A.; Schatz, G. C.; Van Duyne, R. P. *J. Am. Chem. Soc.* **2011**, *133*, 4115–4122.
4. Simtha, S. L.; Gopchandran, K. G.; Smijesh, N.; Philip, R. *Prog. Nat. Sci. Mater. Int.* **2013**, *23*, 36–43.
5. Persaud, I.; Grossman, W. E. L. *J. Raman Spectrosc.* **1993**, *24*, 107–112.
6. Zhang, Y.; Yu, W.; Pei, L.; Lai, K.; Rasco, B. A.; Huang, Y. Food Chem. **2015**, *169*, 80-84.
